# Methodologies of Stigma-Related Research Amongst Men Who Have Sex With Men (MSM) and Transgender People in Asia and the Pacific Low/Middle Income Countries (LMICs): A Scoping Review

**DOI:** 10.3389/frph.2021.688568

**Published:** 2021-10-29

**Authors:** Ni Wayan Septarini, Jacqueline Hendriks, Bruce Maycock, Sharyn Burns

**Affiliations:** ^1^School of Population Health, Curtin University, Perth, WA, Australia; ^2^Department of Community and Preventive Medicine, Faculty of Medicine, Udayana University, Bali, Indonesia; ^3^Collaboration for Evidence, Research and Impact in Public Health, Curtin University, Perth, WA, Australia; ^4^European Center for Environmental and Human Health, College of Medicine and Health, University of Exeter, Exeter, United Kingdom

**Keywords:** culture, discrimination, health, methodology, MSM, stigma, transgender

## Abstract

Much stigma-related research focuses on marginalized populations, including men who have sex with men (MSM) and transgender people. The importance of research in this area is widely recognized, however methodologies and measures vary between studies. This scoping review will collate existing information about how stigma-related research has been conducted in low/middle income countries (LMICs) within the Asia Pacific region, and will compare research designs, sampling frameworks, and measures. Strengths and limitations of these studies will inform recommendations for future stigma-related health research. A methodological framework for scoping studies was applied. Searches of Psych INFO, Scopus, ProQuest, Global Health and PubMed were used to identify articles. Stigma-related research amongst MSM and transgender communities, published between 2010 and 2019 in LMICs within the Asia Pacific region were included. A total of 129 articles based on 123 different studies were included. Of the 129 articles 51.19% (*n* = 66) were quantitative; 44.96% (*n* = 57) were qualitative and 3.88% (*n* = 5) were mixed methods studies. The majority of studies (n = 57; 86.36%) implemented a cross sectional survey. In-depth interviews (*n* = 20, 34.48%) were also common. Only 3.88% of studies utilized mixed-methods design. Non-probabilistic and probabilistic sampling methods were employed in 99.22 and 0.78% of studies respectively. The most common measures used in quantitative studies were the Center for Epidemiological Study on Depression (CES-D) (*n* = 18) and the Self Stigma Scale (SSS) (*n* = 6). Strengths and limitations proposed by researchers included in this review are summarized as lesson learnt and best practices in stigma-related research.

## Introduction

Men who have sex with men (MSM) and transgender communities have been a focus of sexually transmissible infection (STI) prevention in many countries (1–4). These communities may experience a range of social, economic, legal and cultural barriers in accessing physical and mental health interventions especially in low and middle income countries (LMICs) where homosexuality is not legal (5–11). A diverse range of factors contribute to these barriers with stigma and discrimination being significant influences (12). Addressing stigma and discrimination amongst vulnerable communities is challenging.

While there is a body of global research focusing on stigma, the nuances of research with MSM and transgender people are complex. There are a range of factors that can affect the quality of research conducted in this population. In some countries, particularly where homosexuality is not accepted, cultural norms impact stigma and discrimination (6, 13). Therefore, research with MSM or transgender people, especially if Human Immunodeficiency Virus (HIV) infection status is an area of interest, can be problematic. When collecting sensitive data from potentially vulnerable populations it is an ethical imperative that researchers balance potential harms with anticipated benefits.

To date, there is no robust summary or resource detailing methodologies employed for stigma-related research in the context of MSM or transgender populations throughout the Asia-Pacific. Therefore, the focus of this study is to review study designs, sampling frameworks, and specific measures used by researchers from LMICs in this region during the past decade to inform future research. LMICs have been selected as focus in recognition of the different approaches that may be employed in high income countries (HICs) due to differing traditional beliefs and levels of stigma (14). Methodologies undertaken in low-resource settings will also be explored. This review does not intend to determine whether specific methods or measures are more appropriate and accurate than others, as research with marginalized groups are often requires a nuanced approach. This study aims to review/identify research designs, sampling methods and measurements employed in stigma-related research with MSM and transgender communities in LMICs in the Asia-Pacific region and to explore similarities and differences between countries and between population groups (either MSM/transgender or HIV positive/HIV negative). This review addressed three specific questions as follows:

1. What research designs and sampling methods have been used in stigma-related research with MSM and transgender communities in LMICs within the Asia Pacific region?
2. What measures have been used in stigma-related research with MSM and transgender communities in LMICs within the Asia Pacific region?
3. What are the reported limitations, ways to increase strengths and overcome limitations of research methods, sampling methods and measures of the studies focusing on stigma-related research with MSM and transgender communities in LMICs within the Asia Pacific region?

It is intended the review will provide a reference for future research in the area of stigma amongst MSM and transgender people in LMIC in the Asia-Pacific. This review paper has followed the PRISMA extension for scoping reviews (15).

## Materials and Methods

The PRISMA extension for Scoping Reviews (PRISMA-ScR) was employed to provide guidance for this review (15). The five-stage methodological framework for scoping reviews suggested by Arksey and O'Malley (16) was followed. Stages include: (i) identify the research question; (ii) identify relevant studies; (iii) paper selection and screening; (iv) data charting; and (v) collate, summarize and report the results. The following provides a description of each stage:

### Identifying the Research Question

Three specific research questions as described in the introduction were identified.

### Identifying Relevant Studies

Relevant studies from empirical peer-reviewed research articles that examined stigma, discrimination, culture, and health of MSM and transgender communities within the Asia Pacific region-LMICs were identified, retrieved and evaluated.

### Paper Selection and Screening

#### Search Strategy

Eight searches of peer-reviewed manuscripts published from 2010 to 2019 were conducted between May and July 2020 using five databases: Psych INFO, Scopus, ProQuest, Global Health and PubMed. Searches included terms related to (1) “stigma” (2) “discrimination” (3) “culture” (4) “Asia and Pacific countries”, including all countries with this classification based on UNDP in Asia and the Pacific. Supplementary Material 1 of search strategy planner includes all the list of terms that searched in the databases.

#### Inclusion Criteria

Inclusion criteria were peer-review publications which included:

1. an English language abstract;
2. full-text available;
3. research conducted in the Asia-Pacific region (based on the World Health Organization categorization that includes 48 countries) (17).

Exclusion criteria were applied at two stages and included initial screening by title and abstract followed by screening by full text. Exclusion criteria included:

1. Studies conducted in HICs in the Asia Pacific region based on the World Bank categorization (Singapore, Brunei Darussalam, Japan, Korea, Taiwan, Northern Mariana Island, Australia, and New Zealand) (18);
2. Studies not assessing at least one of these categories: health outcomes associated with stigma, discrimination or culture;
3. Editorials, letter to editor, letter, book reviews, systematic/scoping reviews; and
4. Studies including population groups other than MSM and/or transgender people.

Publications were imported into Endnote by NS. Article titles and abstracts were initially screened against the inclusion and exclusion criteria. Two authors, SB and JH were continued to screen the Endnote file based on titles, duplication, and full text availability. All authors discussed final inclusion at this stage.

### Charting the Data

Data were mapped in Excel. The extract data information recorded included: full references, year of publication, country of origin, study design, sampling method(s), number of study participants, study participants, study focus/objective(s), variable(s) measured, measure(s) or scale (s) used, main finding (s), outcome(s), limitation(s), and recommendation(s). Supplementary Material 2 includes all the list of information recorded. Data was extracted by the first author (NS) and cross-checked by all other authors. Following data extraction, it became apparent an individual research project may have resulted in multiple articles. All related articles were included if they met the inclusion/exclusion criteria.

### Summarizing and Reporting the Findings

A “Narrative review” was used to gather similar and different information on all papers to generate a holistic comparison (19). The results presented summary of articles, research methods, and measures that have been used as well as reported limitation of research methodologies.

## Results

**Figure 1:**
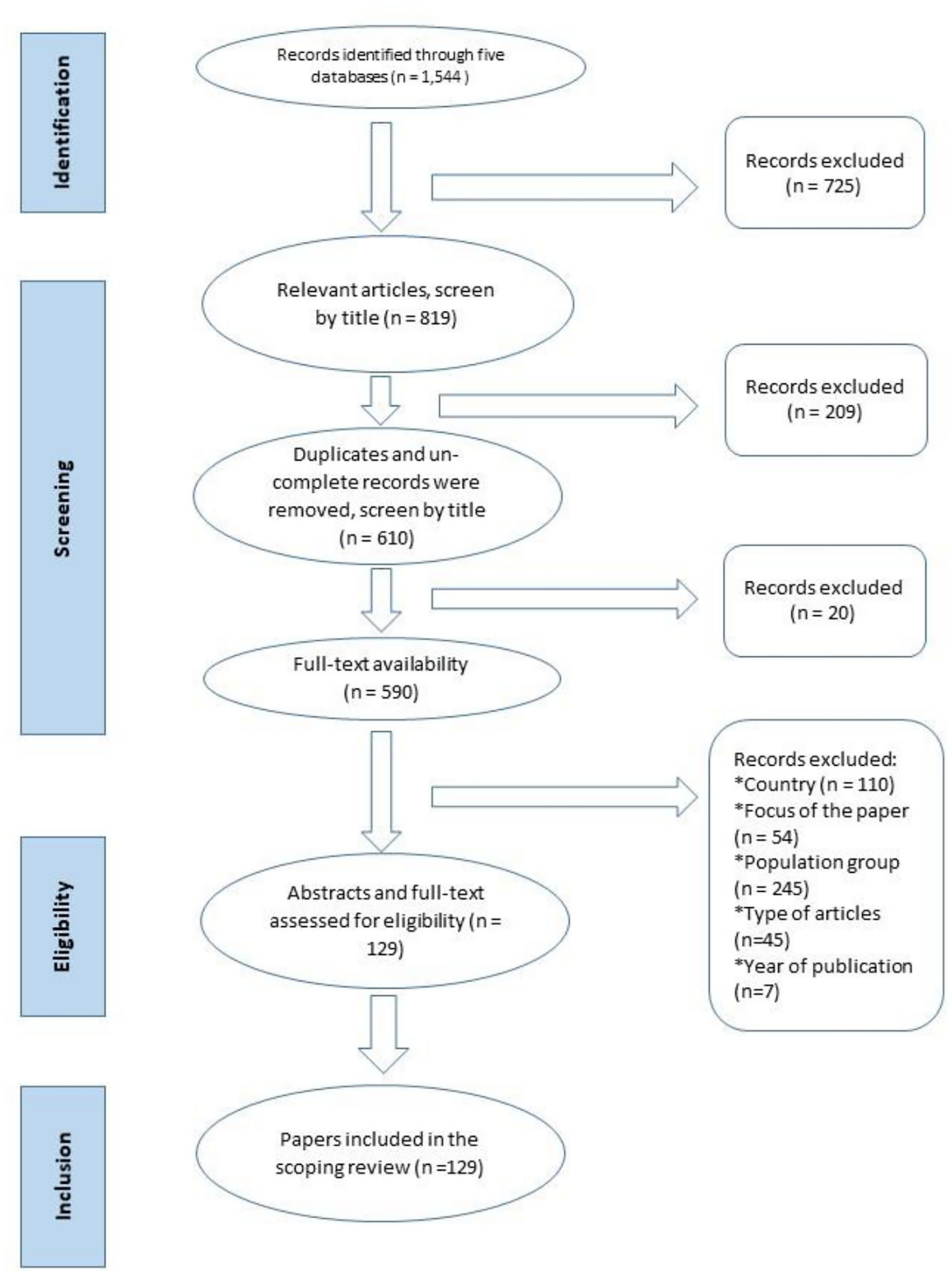
PRISMA flow diagram of scoping review stages based on Arksey and O'Malley (16).

Initially 1,544 potential articles were identified. A total of 816 relevant articles were included after screening based on titles, removing 725 studies. Duplicates were removed (*n* = 209) and full-text was not available for a further 20 articles. Of the 590 remaining publications, articles were removed due to country, population groups, focus of the paper, type of article, and year of publication. A total of 129 articles, based on 123 studies were considered eligible and included in this review. The six articles including studies already described, presented different and important information in each article hence we included in the analysis. Figure 1 details the PRISMA study selection process.

### Articles' Characteristics

**Figure 2:**
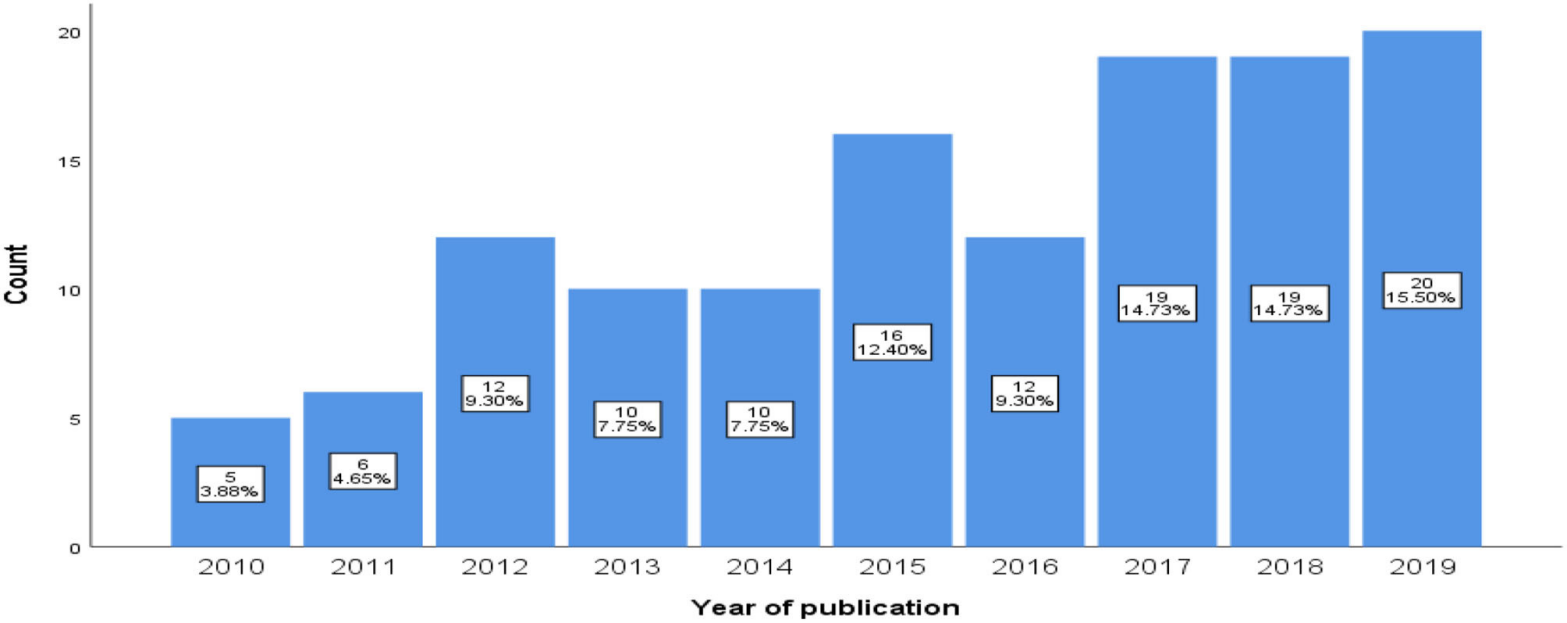
Number of studies and year of publication.

Figure 2 shows that stigma-related research publication in Asia-Pacific LMICs increased during the 2010–2019 period. Stigma-related research among MSM and transgender communities constitutes a growing body of literature, with 66.7% (*n* = 86) of included articles published between 2015 and 2019 compared to around one third (33.3%) from 2010 to 2014.

**Figure 3:**
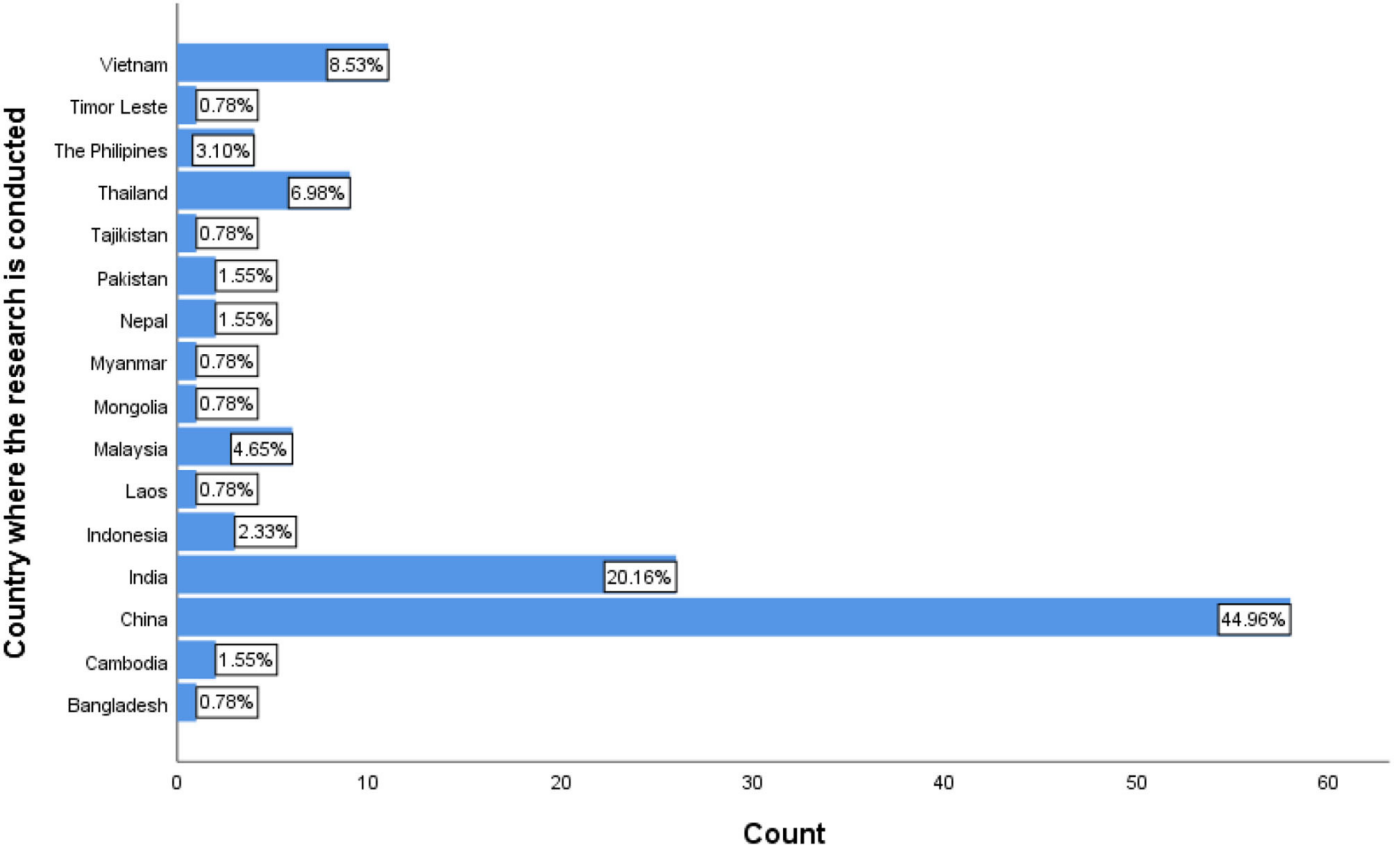
Country where the research was conducted.

Of the 40 LMICs in the Asia Pacific region, research meeting the criteria of this review came from 16 countries. The majority of eligible studies were conducted in China (*n* = 58, 44.96%), India (*n* = 26, 20.16%), Vietnam (*n* = 11, 8.53%), and Thailand (*n* = 9, 6.98%). Forty-one studies (31.81%) were conducted in the South-East Asia region. Figure 3 provides a summary of country of origin.

Table 1 provides a descriptive overview of included study characteristics. Our first research question focused on research designs and sampling methods that have been used in stigma-related research with MSM and transgender communities in LMICs within the Asia Pacific region. Study designs employed quantitative, qualitative and mixed method methodologies. Of the 129 articles included in this review, 66 (51.16%) were quantitative. For most studies (*n* = 57, 86.36%), a cross sectional survey was employed. Other methods (*n* = 9, 13.6%) included trial research, cohort/longitudinal studies, and community-based studies. Qualitative methodology was also common, allowing researchers to explore experiences without drawing any inferences about population-wide trends (21). Common methods employed by the 58 (44.96%) qualitative articles included in-depth interview (*n* = 20, 34.48%) and use of both in-depth interview and focus group discussion (FGD) (*n* = 19c, 32.76). Other methods are described in Table 1. Only five (3.88%) articles employed a mixed methods design, with all of these studies employing quantitative-cross sectional survey and qualitative- in-depth interview or FGD. All mixed method studies in this review utilized this explanatory design. For example, a study conducted in India analyzed a rapid survey (*n* = 247) and subsequently conducted five focus group discussions with married MSM in order to obtain a better understanding of issues like stigma, discrimination, and fear of disclosure in the family (22). Triangulation of the survey data was also conducted by Li et al. (23) when conducting FGD and interviews after the quantitative data collection with different participants. Moreover, Chakrapani et al. (24) conducted in-depth interviews and FGDs to explore barriers to disclosure of HIV status which were initially identified from their quantitative survey.

**Table 1:** Overview of study characteristics.

**Variable**				***N* (%)**	***N* (%)**
**1**	**Study design**				
		Quantitative study			66 (51.16)
			*Cross sectional survey*	57 (86.36)	
			*Trial*	4 (6.06)	
			*Cohort prospective / longitudinal study*	3 (4.54)	
			*Community based study*	2 (3.03)	
			*Total*	66 (100)	
		Qualitative study			58 (44.96)
			*In-depth interview*	20 (34.48)	
			*Semi-structured interview*	3 (5.17)	
			*FGD*	2 (3.45)	
			*In-depth interview & FGD*	19 (32.76)	
			*In-depth interview & Community based study*	1 (1.72)	
			*In-depth interview, FGD, & Community based study*	3 (5.17)	
			*In-depth interview & field work observation*	1 (1.72)	
			*Life story/life history*	3 (5.17)	
			*Case series*	1 (1.72)	
			*Ethnographic study*	2 (3.45)	
			*Ethnographic, participant observation, community based study*	1 (1.72)	
			*Phenomenological study*	1 (1.72)	
			*In-depth interview & phenomenological study*	1 (1.72)	
			*Total*	58 (100)	
		Mixed methods study			5 (3.88)
			*Cross sectional survey & in-depth interview*	1 (20.00)	
			*Cross sectional survey & FGD*	1 (20.00)	
			*Cross sectional survey, in-depth interview, & FGD*	3 (60.00)	
			*Total*	5 (20)	
		Total			129 (20)
**2**	**Sampling method**				
	Probabilistic (random sampling)				1 (0.78)
	Non-probabilistic				128 (99.22)
		*Convenience/Consecutive sampling*		24 (18.75)	
		*Purposive sampling*		27 (21.09)	
		*Snowball sampling/Respondent-driven sampling (RDS)*		24 (18.75)	
		*Multiple methods (using more than one method)*		31 (24.22)	
		*Other methods (i.e., NGOs-driven sampling, theoretical sampling, online recruitment, referral, phone recruitment, ethnographic technique)*		22 (17.19)	
		*Total*		128 (100)	
		Total			129 (100)

While differentiating types of sampling methods, sampling methods are considered to be probabilistic or non-probabilistic (25). The majority of studies (99.22%) in this review employed non-probabilistic sampling methods including convenience/consecutive, purposive, snowballing/respondent-driven sampling (RDS), and multiple sampling methods. Non-probability sampling is useful when random sampling is not possible to conduct, such as when the population is large or hidden (26). Nearly a quarter of studies (18%; *n* = 22) employed multiple sampling methods.

Convenience and consecutive sampling was the most common methods employed in this review. Twenty four articles used convenience/consecutive sampling. Most of these were conducted at health care settings or at places where consultation, testing, and treatment was available (27–32). This method was widely used as random sampling was not usually feasible (8, 33–37) and some helped by non-governmental organizations (NGOs)/community-based organizations (CBOs) staff (12, 38–48). Limitations associated with the use of convenience/consecutive sampling recruitment included that the sampling may not reach all subpopulations and the study may lack the intended diversity (21, 49). However, using recruiters with diverse social-demographic networks may alleviate this (21). Moreover, this sampling framework makes it difficult to achieve a representative sample, thereby limiting the generalizability of the findings (50, 51).

Purposive sampling which enables researchers to recruit participants with specific characteristics or obtain more specific data from a particular group (26), was used by 27 (21.09%) articles in this review. Purposive sampling is commonly used at the explanatory phase of research when the current research is seeking deeper explanation about specific issues. For example, this sampling method was used to understand why transgender women and same-sex-attracted men have intimate sexual relationships with “mane-forte” (straight-identifying men) in Timor-Leste enabling recruitment of specific population groups (52).

MSM and transgender communities may be inter-linked but in hard-to-reach or hidden networks, hence 24 (%) studies employed snowball sampling or RDS technique to reach, recruit, and interview the participants. This non-probability sampling technique is effective when recruiting participants from populations which may be stigmatized and/or hidden (53). Snowball sampling involves researchers asking participants they have recruited to tell their peers about the research (54). This method enables the researcher to get referrals from colleagues/staff working in organizations that may work with MSM and transgender community in addition to referrals from current research participants (55). When using snowball sampling the researchers do not need to know from where participants obtain the referral/information about the study, however this information is important when using RDS (56). RDS was employed in an Integrated Biological and Behavioral Surveillance (IBBS) in Vietnam which recruited 399 MSM (57). “Seeds” who were part of the intended target population were initially identified and recruited by the member(s) of CBOs or NGOs working with the network in order to ensure a broad diversity of socio-demographic characteristics and geographic area representation (57).

There are a number of limitation associated with RDS: firstly, the actual proportion of refusal (non-response bias) cannot be assessed therefore response rates cannot be accurately calculated. Secondly, due to overlapping peer groups, sexual or social networks among the “seed” several recruiters may recruit the same people (57). Thirdly, “seeds selection” bias could occur, which might require a substantial RDS adjustment (57). There are potential concerns regarding selection bias as some groups/networks may remain underrepresented (57). Recruiting participants from disadvantaged populations is not always easy. Studies revealed that MSM and bisexual communities are often reluctant to participate in research due to confidentiality issues (58), resulting in a more non-representative sample.

Thirty-one papers in this review used multiple sampling methods during recruitment to increase representativeness of the research population and generalizability of findings [e.g., (59–61)]. These recruitment methods include outreach work by peer educators or NGO staff, community outreach, venue-based recruitment, and internet advertisements, and web-based recruitment (37, 60). However, these methods were not always successful in recruiting MSM and transgender people as these groups are often difficult to reach, making convenience sampling and RDS a preferred option (62). This method was widely used as random sampling was not usually feasible (36).

### Sampling Framework

The sample size of the studies varied widely Supplementary Material 3. For quantitative studies, sampling size ranged from 10 (63) to 1,375 participants (64, 65), whereas qualitative study samples ranged from 10 (66) to 363 participants (67). Samples for mixed methods studies ranged from 60 (68) to 1,178 participants (10). Participants included MSM, gay men, male sex worker (MSW), mixed (lesbian, gay, bisexual, transgender, LGBTI) identities, transgender women, HIV + MSM, and key informants. Key informants were commonly stakeholders or staff from NGOs or CBOs working with MSM and transgender communities as well as health staff working in MSM-related health services. Young MSM and transgender youth under 18 years were specifically targeted in six studies.

### Variables and Measures

This review explored measures used in stigma-related research with MSM and transgender communities in LMICs within the Asia Pacific region. Included studies used a range of different measures to assess stigma, discrimination and related behaviors, and health. While some researchers developed their own measure, others used previously developed and/or validated measures. When using validated measures, the majority of researchers also calculated Cronbach's Alfa coefficient to determine internal consistency of each measure amongst participants. Cronbach's Alpha coefficient for various measures ranged from 0.60 (questionable) (69) to 0.99 (excellent) (70). Some studies did not report Cronbach's Alfa coefficients.

A range of different measures were employed (see Supplementary Material 4). When measuring depression or depression symptoms, the Center for Epidemiological Studies Depression (CES-D-20) scale was most widely used (71). This measure was used by 12 studies in five different countries including China (41, 55, 72–74), India (48), Nepal (50), Vietnam (75), Cambodia (64, 65). Four studies in this review used this shorted version (CES-D-10) (35, 61, 76, 77). Another short version of this measure, the CES-D 12, was used in China by Nehl et al. (78) and Huang et al. (79).

Researchers suggest to not to employ the CES-D measure as a diagnostic tool, however this measure can be used as the basis for screening and determining the need for further mental health clinical assessment (80). Therefore there is the possibility that data from the CES-D may misrepresent actual prevalence of depression disorders in the population (80). Symptoms of depression might be under-estimated in regions where the behaviors are highly stigmatized (80). Since the measure is a self-report instrument, there is also the possibility of misclassification bias due to social desirability (74).

Stigma among in MSM and transgender communities was measured using a number of self-report tools (Supplementary Material 4). To assess internalized stigma (self-stigma), six studies used either the Self-Stigma Scale (SSS) (81) or the SSS-short version (30, 41, 51, 70, 74). The SSS was developed by Mak et al. (82) after FGDs with groups, comprising LGBT individuals, people with communicable diseases, migrants, and people with mental health problems (82). The original measure, comprises 39 items including affective, behavioral, and cognitive items (83). Each item uses a 6-point scale from strongly disagree [1] to strongly agree [6] (82). However, many studies selected the short nine-item SSS-S version that only captures a subset of affective, behavioral, and cognitive responses (83). The Self-Stigma Scale—Short Form (SSS-S) was used by researchers in China (41, 51), India (23), and the Philippines (70) (*n* = 5).

The Internalized Homophobia Scale, adapted from Meyer (84) was used in four Chinese studies (61, 85–87). Other measures of stigma included the Internalized Shame Scale, also originally developed by Meyer (84) which was used in one Malaysian study [Brown et al. (88)], and the Rosenberg Self Esteem Scale (58) which was used in one study in China (72).

When assessing other types of stigma including gender-conformity, sexual, and HIV-related stigma, research in LMICs in the Asia and Pacific used a range of measures including the Transgender Identity Stigma Scale (TGISS) in India (38, 89, 90); and The Gender Non-Conformity Stigma Scale (GNCSS) also in India (38, 90). The Stigma Consciousness Scale developed by Pinel (91) and the Sexual Compulsivity Scale were used by Xu et al. (87) in study among HIV+ MSM conducted in China.

Research in India used the HIV-related Stigma Assessment scale in research with transgender women (44, 92). In China, several scales to measure HIV-related stigma amongst HIV positive MSM have been used. For example, the Steward's HIV stigma scale was used in research focusing on the relationship between stigma and depression (93), while Li et al. (94) used the Berger's HIV Stigma scale in a HIV-related stigma study. The HIV/AIDS related stigma and discrimination scale was used by Fan et al. (95). Furthermore, the AIDS-related Stigma scale was also used in China on research focus on the impact of homophobia and HIV-related stigma focusing on the uptake of HIV testing (76). While many studies as mentioned employed a validated measure in assessing stigma amongst MSM, a study conducted in China measured three indicators of stigma: internalized, anticipated, and enacted stigma using their own developed instrument (55). In addition to mental health and stigma measures, other measures to assess self-esteem, social support, stress, resilience and coping, and alcohol use were also employed (Supplementary Material 4).

### Reported Limitations

The review also aimed to discuss the reported limitations, ways to increase strengths and overcome limitations of research methods, sampling methods and measures of the studies focusing on stigma-related research with MSM and transgender communities in LMICs within the Asia Pacific region. A number of limitations related to study designs, sample size, and sampling recruitment methods were identified by authors. Nearly 30% of studies (n = 39) discuss the nature of cross-sectional research design as a research limitation. Researchers reported results with caution, especially when making any causal inferences (3, 37, 72, 87, 96). Several studies reported associations rather than the ascertainment of causal relationships or determine the causality of the statistically significant associations between variables (28, 29, 38, 62, 97). This design also undermines the ability to draw conclusions about causality on the evidence (41, 50, 77, 81, 89, 98). However, researchers acknowledge the possibility of other directional associations between the variables of interests in the study (30). Moreover, in order to mitigate the use of cross-sectional design, researcher suggests to add some qualitative research insights (49).

Nearly 10% (*n* = 12) of studies discussed the nature of qualitative research as a study limitation. Data saturation is a critical consideration in qualitative research. In order to reach data saturation, certain number of participants are needed. Studies conducted in China reported since the sample size was small, and authors identified saturation was not achieved [*n* = 10 (63) and *n* = 14 (5)]. Other research analyzed only 26% of all data collected (39 interview transcripts/149 interviews) due to saturation in themes (99).

The majority of studies were either exploratory or explanatory with the majority (n=82, 63.57%) indicating study findings were only applicable to certain population groups and could not be generalized (20). Research focusing on a particular group or network, for example research with HIV positive MSM, transgender women, or male sex workers was only applicable in these settings (100).

Moreover, some studies were unable to make generalizable conclusions due to recruitment methods. For example, recruitment *via* online survey (87) or through a medical center (101), could not generalize findings to the wider population as not all members of the community may have had access to the internet or attended the medical center (87, 101, 102). Furthermore, most MSM and transgender people living with HIV/AIDS are difficult to reach by offline sampling methods because of the dual stigma and discrimination toward HIV infection and homosexuality (60).

Research using other types of non-probability sampling methods, such as purposive, convenience, and snow-ball sampling techniques also provided similar statements about restrictions on the generalizability of research findings (29, 33, 34, 38, 50, 51, 89, 101, 103–107). As an example, even though participants in Wei et al.'s (2014) study were diverse based on sociodemographic characteristics, the qualitative findings could not be generalized because participants were recruited *via* convenience sample (107). Geographic location was also cited as a reason that findings were not representative of the broader community (43, 65, 75, 102). Convenience sampling was used due to stigma and prejudice surrounding MSM and transgender communities and related research topics which may have impacted recruitment using other methods (43, 108). Some researchers also expressed caution when using research findings in other regions of the study country as the pattern of variables being measured may differ and the access to health services, and cultural beliefs may differ (55, 58).

Beside limitations related to study design and generalizability, limitations also exist around systematic error/bias. Four types of bias were commonly identified: included socially desirability (*n* = 23, 17.83%); self-report/response (*n* = 31, 24.03%); participation (*n* = 40, 31.01%); and information/recall bias (*n* = 8, 6.20%). Studies that focus on sensitive issues or gather data that may be viewed as “illegal” or unacceptable by family, society or the law, must consider social desirability and self-report bias (35, 37, 42, 65, 89, 106, 109). Participants in these studies may feel ashamed and/or uncomfortable to express their attitudes and behaviors during face-to-face interviews which include sensitive questions (37, 110). Participants' self-report might also be affected by their sociocultural background (77). One study suggested that self-report bias might be reduced by selecting interviewers that were experienced and well known in the study site (80). Self-report bias may under-estimate the true prevalence of particular attitudes or behaviors due to under-reporting of issues such as drug abuse (42), unprotected sex (37), and sexual violence (28, 37, 98). However, self-report bias can be minimized by certain activities such as building a good rapport with the proposed participants, providing additional material and details about the benefit of the study, ensuring confidentiality, providing comprehensive explanations about the topic of the research in addition to providing opportunity to ask questions in a safe environment (42, 111). Another way to increase the reliability of self-report data and to reduce socially desirable bias is by employing Audio-Computer-Assisted Self-Interview (ACASI) or other computer-assisted questionnaires to collect behavioral data (77, 112). Another study suggested that participants' discomfort maybe diminished by conducting interviews at MSM-friendly venues and by efforts of well-trained and experienced research team including peers as data collectors (49).

Studies addressing stigma may also be prone to participation bias. Forty studies (31.01%) of in this review acknowledged participation bias. Those with the strongest stigma concerns are likely to be underrepresented because they would be the most concerned about leaving contact information for follow-up in a longitudinal study (55). This concern can be managed by allowing the participants to provide pseudonyms and allowing them to provide less identifiable forms of contact information, for example a social media platform address or a cruising site (55, 73). Studies also revealed that the inclusion of incentive also influenced participation, with a person more likely to participate in research if a financial incentive was provided, especially for those come from low socioeconomic status (28). This type of bias which is sometimes called self-selection bias, may exist when convenience sampling is used when recruiting participants, for example studies conducted in China acknowledged the possibility of self-selection bias due to respondents recruited *via* the internet (36, 74).

Recall bias, where participants fail to accurately report their past actions, is a type of information bias which influences the validity of information gained from the participants (57) and may influence the magnitude of associations between variables (98). Eight (6.2%) studies acknowledged information/recall bias as a limitation. For example, recall bias was identified in a longitudinal study with a relatively long spacing (e.g., 6 months) between data collection time points, particularly when measuring mental health outcomes that typically only consider the previous few weeks (73). It was also evident when data relied on retrospective self-reports within surveys (110) or in-depth interviews (24). For example, questions about condom and lubricant use over a previous 6 or 12 months period are likely to be open to recall bias (95, 113).

## Discussion

This scoping review aimed to review study design and methods, measure and reported limitations on studies focusing on stigma-related research with MSM and transgender communities in LMICs within the Asia Pacific region. Research evidence in this area has significantly increased during the period 2010–2019. The majority of research was conducted in China and India, which are the two largest countries in this region. Included studies were concerned with different influences of stigma, with different methodologies. Study designs which included quantitative, qualitative, and mixed-methods designs and were dependent on the research focus target population, and setting. Cross sectional survey was the most popular, which is likely associated with the feasibility and suitability of this design for accessing “hidden populations” which have been found to be difficult to follow up in longitudinal study or trial (81). However, some studies recommended that longitudinal research with adequate sample size and probability-based sampling procedures is likely to better support testing complex models and causalities or in order to verify the results (37, 38, 72). Intervention-based research may also provide an alternate option to conduct to measure the impact of interventions on attitudes or behaviors (81). Despite the potential of longitudinal design, a Chinese study found use of this design did not provide a definite causal interpretation (55). Two cross sectional design papers in this review described the baseline survey of an intervention study (68, 93). The cross sectional survey was most often selected when the researchers sought to explore stigma, attitudes, behaviors, and health outcome of marginalized groups using specific previously validated measures.

Qualitative study design is another option for conducting research in MSM and transgender communities. In-depth interview was the most commonly used qualitative methodology employed, which may be associated with the confidentiality this method affords in comparison to FGDs. However, in order to obtain different perspectives from various participants, many researchers employed multiple data collection methods (Table 1). Qualitative designs allowed researchers to explore phenomena and issues in greater depth. Qualitative research method enables exploration which generalization of results is not required (114).

Qualitative design was also used to triangulate quantitative data from surveys conducted in a mixed-methods design (33, 68, 115). For studies with small sample sizes, triangulation may increase validity. For example, a study in India employed a mixed-methods design which included survey, in-depth interview, and focus groups, and data sources were triangulated (MSM and transgender) to investigate sexual risk behaviors and HIV status disclosure amongst HIV positive MSM and transgender people (24). Exploratory sequential mixed methods design occurs when the qualitative data collection and analysis builds or develops a quantitative instrument or quantitative intervention (116). This method was not employed by any paper in this review.

A wide variety (*n* = 49) of measures were used to measure mental health and stigma across 123 studies. The CES-D (27) was commonly used to measure depressive symptoms and the SSS (81) or SSS-short version (70) to measure internalized stigma. This review did not compare measures given the diversity in population groups, settings and variables explored. Internal consistency in quantitative studies of measures was not cited in 14 studies. However, when reported, Cronbach's alpha coefficient of the measure in their sample felt between 0.60 and 0.99. However there is debate around the efficacy of using Cronbach's alpha to measure the internal consistency of measures. While Schmitt (117) argued that presenting alpha information is not sufficient and inter-correlations and corrected inter-correlations should also be reported. Further, there level of acceptability is also contested with measures reporting (by conventional standards) low levels of alpha continuing to be useful in some cases (117). In contrast Heo et al. (118) conclude that cross sectional and longitudinal research should use instruments with greater Cronbach's alpha since they have smaller measurement error and greater statistical power. Enhancing Cronbach's alpha of the instrument when questions are parallel targeting a unidimensional construct is also needed and can be done by developing a set of highly correlated items but not by excessively increasing the number of items with insufficient inter-item correlations (118).

Some researchers discussed ways to overcome the limitation of their study design. This review summarized considerations around conducting stigma-related research. This includes the selection of study designs, sample size, generalizability, measures used, and the possibility of systematic error or bias (Table 2). These considerations are similar to conducting research in other marginalized populations such as female sex workers (122), and research in harm reduction among people who inject drugs (123). Researchers did not discuss limitations in relation to sampling collection methods, as many MSM and transgender in LMICs within the Asia Pacific region are hidden and difficult to reach due to prejudice and legality concerns. This conclusion is similar to a study conducted in the US, which used multiple methods to recruit 6,456 transgender participants (124). Choosing suitable sampling methods is important when conducting research in disadvantaged populations. Some considerations proposed by studies included difficulties in reaching the target population, the sensitive nature of research topics, and time allocation. Since MSM and transgender populations are mostly hidden and their sexual behaviors deemed illegal in some Asia Pacific-LMICs, researchers commonly chose multiple recruitment methods, in order to achieve the desired sample size and recruit a diverse sample [e.g., (125)].

**Table 2:** Limitations of MSM and transgender studies.

**Area of limitations**		**Number of studies reported the limitations (%)**	**Example of studies**
Study design	Quantitative-cross sectional survey	38 (29.46)	(30, 37, 76)
	Qualitative	12(9.30)	(119, 120)
Sample size/generalizability		82 (63.57)	(72, 97, 121)
Measure/Scale used		31 (24.03)	(79, 92)
Bias	Socially desirable	23(17.83)	(28, 105)
	Self-report/response	31 (24.03)	(36, 98)
	Participation	40 (31.01)	(88, 104)
	Information/recall	8 (6.20)	(57, 95)

*One study may reported more than one limitations*.

This review is not without its own limitations. It is possible that more explanation could be included if authors had been personally approached to provide information on the methodologies chosen. Future reviews of stigma-related research in MSM and transgender population would also benefit from using available validated tools to critically appraise the quality of included studies. Moreover, other factors influencing the quality of research should also be assessed, including survey translation, response rate, data saturation, and validation of overall instruments. This would assist cross country and population comparisons. This scoping review is also only included studies in English language and did not include “gray literature” or doctoral theses. Finally, this review is not a systematic literature review, therefore, we did not assess or exclude papers based on their quality.

## Data Availability Statement

The original contributions presented in the study are included in the article/Supplementary Material, further inquiries can be directed to the corresponding author.

## Author Contributions

NS and SB: conceptualized the paper. NS and JH: conducted data curation. NS, SB, and JH: conducted the formal analysis. All authors drafted, reviewed, and edited the paper.

## Conflict of Interest

The authors declare that the research was conducted in the absence of any commercial or financial relationships that could be construed as a potential conflict of interest.

## Publisher's Note

All claims expressed in this article are solely those of the authors and do not necessarily represent those of their affiliated organizations, or those of the publisher, the editors and the reviewers. Any product that may be evaluated in this article, or claim that may be made by its manufacturer, is not guaranteed or endorsed by the publisher.
