# Supplementary material for: Methodologies of Stigma-Related Research Amongst Men Who Have Sex With Men (MSM) and Transgender People in Asia and the Pacific Low/Middle Income Countries (LMICs): A Scoping Review
Source: Front Reprod Health. 2021 Oct 29;3:688568. doi: 10.3389/frph.2021.688568 (PMC9580832; doi:10.3389/frph.2021.688568)
Supplement: Supplementary file 1 [file Table_1.DOCX]

***Table 1***

**Search Strategy Planner**

The intended title of the review is

**Stigma, discrimination, culture, and health amongst MSM and transgender in Asia and the Pacific: a scoping review of the quantitative and qualitative evidence.**

Scopus

(HIV OR AIDS OR STI OR “sexually transmitted infection” OR syphilis OR gonorrhoea OR chlamydia  OR HPV OR “human papilloma virus” OR Trichomonas OR Herpes OR Wart OR “Hepatitis B”   AND

(stigma OR discrimination) AND

culture AND

(MSM OR “men who have sex with men” OR transgender OR waria) AND

(China OR India OR Indonesia OR Pakistan OR Bangladesh OR Japan OR Philippines OR Vietnam OR Thailand OR Myanmar OR “South Korea” OR Malaysia OR Nepal OR “North Korea” OR Australia OR Taiwan OR “Sri Lanka” OR Cambodia OR “Papua New Guinea” OR Laos OR Singapore OR “New Zealand” OR Mongolia OR “Timor Leste” OR Fiji OR Bhutan OR “Solomon Islands”  OR Maldives OR Brunei OR Vanuatu OR “New Caledonia” OR “French Polynesia” OR Samoa OR Guam OR Kiribati OR Micronesia OR Tonga OR “Marshall Islands” OR “Northern Mariana Islands” OR “American Samoa” OR Palau OR “Cook Islands” OR Tuvalu OR “Wallis and Futuna” OR Nauru OR Niue OR Tokelau)

In Articles title, Abstract, Keywords

Further:

Year: 2010 to 2019

Document type:  Article

Access type: Open access and other

Subject area excludes: Dentistry; Economics, econometrics and finance; Biochemistry, genetics and molecular biology; Business, management and accounting; environmental science; Pharmacology, toxicology, and pharmaceutics; computer science; energy; engineering; mathematics.

Keyword exclude: female

Result: 303 documents  192 title relevant based on the title

PUBMED

(HIV OR AIDS OR STI OR “sexually transmitted infection” OR syphilis OR gonorrhoea OR chlamydia  OR HPV OR “human papilloma virus” OR Trichomonas OR Herpes OR Wart OR “Hepatitis B”   AND

(stigma OR discrimination) AND

culture AND

(MSM OR “men who have sex with men” OR transgender OR waria) AND

(China OR India OR Indonesia OR Pakistan OR Bangladesh OR Japan OR Philippines OR Vietnam OR Thailand OR Myanmar OR “South Korea” OR Malaysia OR Nepal OR “North Korea” OR Australia OR Taiwan OR “Sri Lanka” OR Cambodia OR “Papua New Guinea” OR Laos OR Singapore OR “New Zealand” OR Mongolia OR “Timor Leste” OR Fiji OR Bhutan OR “Solomon Islands”  OR Maldives OR Brunei OR Vanuatu OR “New Caledonia” OR “French Polynesia” OR Samoa OR Guam OR Kiribati OR Micronesia OR Tonga OR “Marshall Islands” OR “Northern Mariana Islands” OR “American Samoa” OR Palau OR “Cook Islands” OR Tuvalu OR “Wallis and Futuna” OR Nauru OR Niue OR Tokelau)

Result: 71  43 relevant articles based on the title

PROQUEST

 noft(stigma or discriminat* or stereotyp* or homophob*

) AND noft(culture or norm or value) AND noft(syphilis or gonorrhoea or chlamydia or trichomon* or genital herpes or genital wart* or hepatitis B

or HPV or human papilloma virus or STI or sexually transmitted infection* or HIV or human immunodeficiency virus* or AIDS or acquired immunodeficiency syndrome

) AND noft(men who have sex with men or MSM or transgender

) AND noft(Asia OR Pacific OR

China OR India OR Indonesia OR Pakistan OR Bangladesh OR Japan OR Philippines OR Vietnam OR Thailand OR Myanmar OR South Korea OR Malaysia OR Nepal OR North Korea OR Australia OR Taiwan OR Sri Lanka OR Cambodia OR Papua New Guinea OR Laos OR Singapore OR New Zealand OR Mongolia OR Timor Leste OR Fiji OR Bhutan OR Solomon Islands OR Maldives OR Brunei OR Vanuatu OR New Caledonia OR French Polynesia OR Samoa OR Guam OR Kiribati OR Micronesia OR Tonga OR Marshall Islands OR Northern Mariana Islands OR American Samoa OR Palau OR Cook Islands OR Tuvalu OR Nauru OR Niue OR Tokelau OR Wallis OR Futuna)

Limit search: 01-01-2010 ----31-12-2019

Result: 65 peer reviewed articles 🡺 57 relevant articles based on the title

21 July 2020

Global Health

Advanced search

Search Strategy:

1. (stigma or discriminat* stereotyp* homophob* culture).mp. [mp=abstract, title, original title, broad terms, heading words, identifiers, cabicodes]
2. (syphilis or gonorrhoea or chlamydia or trichomon* or genital herpes or genital wart* or hepatitis B or HPV or human papilloma virus or STI or sexually transmitted infection* or HIV or human immunodeficiency virus*).mp. [mp=abstract, title, original title, broad terms, heading words, identifiers, cabicodes]
3. 1 and 2
4. (young adults or men or adolescents or homosexuality or acquired immune deficiency syndrome or HIV infections or sex workers or behaviour or human immunodeficiency viruses or risk behaviour or sexually transmitted diseases or disease transmission or men who have sex with men or sexual behaviour or health care or anal intercourse or disease prevention).sh. or man.od.
5. 3 and 4
6. (Asia or Pacific or China or India or Indonesia or Pakistan or Bangladesh or Japan or Philippines or Vietnam or Thailand or Myanmar or South Korea or Malaysia or Nepal or North Korea or Australia or Taiwan or Sri Lanka or Cambodia or Papua New Guinea or Laos or Singapore or New Zealand or Mongolia or Timor Leste or Fiji or Bhutan or Solomon Islands or Maldives or Brunei or Vanuatu or New Caledonia or French Polynesia or Samoa or Guam or Kiribati or Micronesia or Tonga or Marshall Islands or Northern Mariana Islands or American Samoa or Palau or Cook Islands or Tuvalu or Nauru or Niue or Tokelau or Wallis or Futuna).mp. [mp=abstract, title, original title, broad terms, heading words, identifiers, cabicodes]
7. 5 and 6
8. limit 7 to (english language and yr="2010 - 2019")

Result: 778 articles 🡺 345 relevant based on the title

21 July 2020

APA PsycInfo

Advanced Search

Search strategy:

1. (stigma or discriminat* stereotyp* homophob* culture).mp. [mp=title, abstract, heading word, table of contents, key concepts, original title, tests & measures, mesh]
2. (syphilis or gonorrhoea or chlamydia or trichomon* or genital herpes or genital wart* or hepatitis B or HPV or human papilloma virus or STI or sexually transmitted infection* or HIV or human immunodeficiency virus*).mp. [mp=title, abstract, heading word, table of contents, key concepts, original title, tests & measures, mesh]
3. 1 and 2
4. (young adults or men or adolescents or homosexuality or acquired immune deficiency syndrome or HIV infections or sex workers or behaviour or human immunodeficiency viruses or risk behaviour or sexually transmitted diseases or disease transmission or men who have sex with men or sexual behaviour or health care or anal intercourse or disease prevention).mp. [mp=title, abstract, heading word, table of contents, key concepts, original title, tests & measures, mesh]
5. 3 and 4
6. (Asia or Pacific or China or India or Indonesia or Pakistan or Bangladesh or Japan or Philippines or Vietnam or Thailand or Myanmar or South Korea or Malaysia or Nepal or North Korea or Australia or Taiwan or Sri Lanka or Cambodia or Papua New Guinea or Laos or Singapore or New Zealand or Mongolia or Timor Leste or Fiji or Bhutan or Solomon Islands or Maldives or Brunei or Vanuatu or New Caledonia or French Polynesia or Samoa or Guam or Kiribati or Micronesia or Tonga or Marshall Islands or Northern Mariana Islands or American Samoa or Palau or Cook Islands or Tuvalu or Nauru or Niue or Tokelau or Wallis or Futuna).mp. [mp=title, abstract, heading word, table of contents, key concepts, original title, tests & measures, mesh]
7. 5 and 6
8. Limit 7 to (English language and yr=”2010-2019”)

Result: 327 articles 🡺 197 relevant based on the title

Total result: 1,544 articles 🡺 819 relevant articles based on the title 🡺 doubled and un-completed (n=170) 🡺 649 articles 🡺 Full- text available: 629 articles

**Key terms and subject headings applied**

**Medline/Pubmed, Psycinfo, Global Health, Scopus(or Web of Science), Proquest(or ScienceDirect), Google Scholar**

**AND**

**OR**

**AND**

**AND**

|  | **CONCEPT 1** | **CONCEPT 2** | **CONCEPT 3** | **CONCEPT 4** |
| --- | --- | --- | --- | --- |
| **Key Terms** | stigma  discriminat*  stereotyp*  homophob*  culture | syphilis  gonorrhoea  chlamydia  trichomon*  genital herpes  genital wart*  hepatitis B  HPV  human papilloma virus  STI  sexually transmitted infection*  HIV  human immunodeficiency virus*  AIDS  acquired immunodeficiency syndrome | men who have sex with men  MSM  transgender | Asia OR Pacific OR  China OR India OR Indonesia OR Pakistan OR Bangladesh OR Japan OR Philippines OR Vietnam OR Thailand OR Myanmar OR South Korea OR Malaysia OR Nepal OR North Korea OR Australia OR Taiwan OR Sri Lanka OR Cambodia OR Papua New Guinea OR Laos OR Singapore OR New Zealand OR Mongolia OR Timor Leste OR Fiji OR Bhutan OR Solomon Islands OR Maldives OR Brunei OR Vanuatu OR New Caledonia OR French Polynesia OR Samoa OR Guam OR Kiribati OR Micronesia OR Tonga OR Marshall Islands OR Northern Mariana Islands OR American Samoa OR Palau OR Cook Islands OR Tuvalu OR Nauru OR Niue OR Tokelau OR Wallis or Futuna |
| **Subject Headings**  **Medline - MESH** | Social Stigma/  Social Discrimination/  Homophobia/  Stereotyping/  Culture/ | exp Sexually Transmitted Diseases/  [includes HIV, etc]  Papillomavirus Infections/ | exp Sexual and Gender Minorities/  [includes transgender]  Homosexuality, Male/  Gender Identity/  Sexual Behavior/  Sexuality/ | exp Asia/  [or select specific regions]  exp Australasia/  [or exp Oceania/] |
