# Supplementary material for: Methodologies of Stigma-Related Research Amongst Men Who Have Sex With Men (MSM) and Transgender People in Asia and the Pacific Low/Middle Income Countries (LMICs): A Scoping Review
Source: Front Reprod Health. 2021 Oct 29;3:688568. doi: 10.3389/frph.2021.688568 (PMC9580832; doi:10.3389/frph.2021.688568)
Supplement: Supplementary file 3 [file Table_3.DOCX]

Table 3

Table 3. Included study (n = 129) with key characteristics and sampling framework

| **Key characteristics** | | | | **Sampling framework** | | |
| --- | --- | --- | --- | --- | --- | --- |
| **No** | **Author(s)** | **Country of study** | **Study design** | **Sampling methods** | **Sample size (total)** | **Study participants** |
| 1 | Abdullah et al., 2012 | Pakistan | Qualitative-in-depth interview, FGD | Purposive | 36 | Transgender |
| 2 | Adia et al., 2018 | The Philippines | Qualitative-interview | Convenience | 21 | 15 HIV+ MSM & 6 NGOs staffs |
| 3 | Anand et al., 2017 | Thailand | Qualitative-in-depth interview | Phone recruitment | 18 | 16 young HIV+ MSM & 2 young HIV+ transgender women (aged 16-24 y.o.) |
| 4 | Anjana et al., 2019 | India | Quantitative-trial | Multiple methods | 247 | MSM |
| 5 | Aparna et al., 2018 | Viet Nam | Quantitative-cross sectional survey | Snowball | 205 | MSM |
| 6 | Argento et al., 2011 | India | Qualitative-in-depth interview, FGD | Purposive | 46 | 34 Sex workers & 12 stakeholders |
| 7 | Azyei et al., 2014 | Mongolia | Quantitative-cross sectional survey | RDS | 200 | MSM |
| 8 | Berry et al., 2013 | Viet Nam | Qualitative -in-depth interview | Purposive | 34 | 30 MSM & 4 key informants |
| 9 | Bowring et al., 2015 | Laos | Qualitative-in-depth interview, FGD | Multiple methods | 31 | 17 bisexual men & 14 key experts |
| 10 | Brown, Low, Tai, & Tong, 2016 | Malaysia | Quantitative- cross sectional survey | Online recruitment | 234 | gay men |
| 11 | Bui et al., 2017 | Viet Nam | Qualitative-in-depth interview | Snowball sampling | 35 | MSM |
| 12 | Burch, Hart, & Lim, 2018 | Malaysia | Qualitative-semi-structured interview | Multiple methods | 24 | Young MSM (aged 18-25 y.o.) |
| 13 | Chakrapani, Boyce, et al., 2013 | India | Qualitative-in-depth interview, FGD | Multiple methods | 121 | 105 MSM & 16 key informants |
| 14 | Chakrapani, Kaur, Newman, Mittal, & Kumar, 2019 | India | Qualitative-in-depth interview, FGD | Purposive sampling | 37 | 30 MSM & 7 key informants |
| 15 | Chakrapani, Newman, Shunmugam, & Dubrow, 2011 | India | Qualitative-FGD, interview | Purposive sampling | 38 | 34 HIV+ transgender & 4 community leaders |
| 16 | Chakrapani, Newman, et al., 2015 | India | Qualitative-FGD, interview | Purposive sampling | 71 | 68 MSM & 14 community leaders |
| 17 | Chakrapani, Newman, Singhal, Jerajani, & Shunmugam, 2012 | India | Qualitative-Community based study, in-depth interview, FGD | Purposive sampling | 82 | 68 MSM & 14 key informants |
| 18 | Chakrapani, Newman, Singhal, Nelson, & Shunmugam, 2013 | India | Qualitative-Community based study, in-depth interview, FGD | Purposive sampling | 82 | 68 MSM & 14 key informants |
| 19 | Chakrapani, Shunmugam, Newman, Kershaw, & Dubrow, 2015 | India | Mixed-methods study-cross sectional survey, FGD, in-depth interview | Multiple methods | 136 | HIV+ MSM & transgender |
| 20 | Chakrapani et al., 2017 | India | Quantitative-cross sectional survey | Convenience sampling | 600 | 300 Transgender women & 300 MSM |
| 21 | Chakrapani, Willie, Shunmugam, & Kershaw, 2019 | India | Quantitative-cross sectional survey | NGOs-driven sampling | 300 | Transgender women |
| 22 | Chan & Mak, 2019 | China | Quantitative-cross sectional survey | Consecutive sampling/referral | 206 | HIV+ MSM |
| 23 | Chen et al., 2012 | China | Quantitative-cross sectional survey | Snowball sampling | 714 | MSM |
| 24 | K. H. Choi, Steward, Miège, Hudes, & Gregorich, 2016 | China | Quantitative-Longitudinal data (baseline, 6,& 12 months) | Snowball sampling | 493 | MSM |
| 25 | K.-H. Choi, Steward, Miege, & Gregorich, 2017 | China | Quantitative-Longitudinal data (baseline, 6,& 12 months) | Snowball sampling | 493 | MSM |
| 26 | Chong, Mak, Tam, Zhu, & Chung, 2017 | China | Quantitative-cross sectional survey | Consecutive sampling/referral | 100 | HIV+ MSM |
| 27 | Chow et al., 2013 | China | Qualitative-in-depth interview | Purposive sampling | 15 | MSM |
| 28 | Closson et al., 2015 | Vietnam | Qualitative-in-depth interview | NGOs-driven sampling | 23 | Male sex workers (MSW) |
| 29 | Davis, Miles, & Quinley Iii, 2019 | Thailand | Mixed-methods-cross sectional survey, interview | Multiple methods | 60 | Transgender sex workers |
| 30 | de Lind van Wijngaarden, Schunter, & Iqbal, 2013 | Pakistan | Qualitative-life histories | NGOs-driven sampling | 10 | Young feminised men |
| 31 | Deuba et al., 2013 | Nepal | Quantitative- cross sectional survey | Snowball sampling | 339 | MSM |
| 32 | Du et al., 2018 | China | Quantitative study | NGOs-driven sampling | 321 | HIV+MSM |
| 33 | Dutta, Khan, & Lorway, 2019 | India | Qualitative- ethnographic study | Multiple methods | 41 | Transgender women (jogappas) |
| 34 | Fan et al., 2016 | China | Quantitative-cross sectional survey | Multiple methods | 391 | MSM |
| 35 | Felix, 2014 | Malaysia | Qualitative-in-depth interview | Snowball sampling | 33 | Gay men |
| 36 | Feng et al., 2010 | China | Qualitative-in-depth interview, FGD | NGOs-driven sampling | 43 | MSM |
| 37 | Ganju & Saggurti, 2017 | India | Qualitative-in-depth interview | Multiple methods | 68 | Transgender sex workers |
| 38 | Gibson et al., 2016 | Malaysia | Qualitative-in-depth interview | Multiple methods | 21 | Transgender sex workers |
| 39 | Gu, Lau, & Tsui, 2011 | China | Quantitative-cross sectional survey | Multiple methods | 577 | MSM |
| 40 | Guadamuz, Goldsamt, & Boonmongkon, 2015 | Thailand | Qualitative-community based study, in-depth interview, FGD | NGOs-driven sampling | 33 | Younger than 18 years MSM and parents |
| 41 | Guo, Li, Liu, Jiang, & Tu, 2014 | China | Quantitative-cross sectional survey | Multiple methods | 307 | migrant MSM |
| 42 | Ha, Risser, Ross, Huynh, & Nguyen, 2015 | Vietnam | Quantitative-cross sectional survey | RDS | 451 | MSM |
| 43 | He et al., 2017 | China | Qualitative-in-depth interview | NGOs-driven sampling | 61 | Gay men |
| 44 | Huang et al., 2012 | China | Quantitative-cross sectional survey | RDS | 397 | MSM |
| 45 | Hussain, Kulshreshtha, & Yadav, 2018 | India | Quantitative-cross sectional survey | Convenience sampling | 277 | MSM |
| 46 | Ibragimov & Wong, 2018 | Tajikistan | Qualitative-in-depth interview, FGD | Purposive sampling | 21 | Gay & bisexual men |
| 47 | Jackson et al., 2012 | China | Quantitative-cross sectional survey | Multiple methods | 570 | MSM |
| 48 | Khan et al., 2019 | Bangladesh | Qualitative-in-depth interview, FGD | Multiple methods | 69 | MSM, MSW, & transgender women |
| 49 | Koo et al., 2014 | China | Qualitative-in-depth interview | Purposive sampling | 15 | MSM |
| 50 | Lazuardi et al., 2019 | Indonesia | Qualitative-in-depth interview, FGD | Purposive sampling | 54 | MSM |
| 51 | D. H. Li et al., 2017 | India | Mixed-methods-cross sectional survey, in-depth interview, FGD | Snowball sampling | 459 | MSM & transgender women & 10 health care provider |
| 52 | H. Li, Lau, Holroyd, & Yi, 2010 | China | Qualitative-ethnographic study | Theoretical sampling method | 17 | MSM |
| 53 | H. Li et al., 2017 | China | Qualitative-in-depth interview | Purposive sampling | 21 | HIV+ MSM |
| 54 | H. H. Li, Holroyd, Lau, & Li, 2015 | China | Qualitative-life story interview | Purposive sampling | 31 | HIV+ MSM |
| 55 | H. H. Li, Holroyd, Li, & Lau, 2015 | China | Qualitative-in-depth interview | Purposive sampling | 31 | HIV+ MSM |
| 56 | J. Li et al., 2016 | China | Quantitative-cross sectional survey | Phone recruitment | 321 | HIV + MSM |
| 57 | J. Li, Mo, Wu, & Lau, 2017 | China | Quantitative-cross sectional survey | Multiple methods | 321 | HIV+ MSM |
| 58 | X. Li, Lu, Ma, et al., 2012 | China | Quantitative-cross sectional survey | RDS | 500 | MSM |
| 59 | X. Li, Lu, Raymond, et al., 2012 | China | Quantitative-cross sectional survey | RDS | 500 | MSM |
| 60 | X. Li et al., 2014 | China | Quantitative-cross sectional survey | RDS | 992 | MSM |
| 61 | Z. Li, Hsieh, Morano, & Sheng, 2016 | China | Quantitative-cross sectional survey | Convenience sampling | 266 | HIV+ MSM |
| 62 | MeiZhen Liao et al., 2014 | China | Quantitative-cross sectional survey | Multiple methods | 1,230 | MSM |
| 63 | M. Liao et al., 2015 | China | Quantitative-cross sectional survey | Multiple methods | 1,230 | MSM |
| 64 | Lim et al., 2019 | Malaysia | Qualitative-in-depth interview | Convenience sampling | 20 | HIV+ MSM |
| 65 | Lin et al., 2016 | China | Qualitative-in-depth interview | Consecutive sampling | 57 | HIV+ MSM |
| 66 | Liow, Fazli Khalaf, Mohammad Ameeruddin, & Foong, 2017 | Malaysia | Qualitative-phenomenological study | Multiple methods | 12 | Homosexual Men |
| 67 | Liu et al., 2018 | China | Quantitative-cross sectional survey | RDS | 807 | MSM |
| 68 | Yu Liu et al., 2015 | China | Quantitative-cross sectional survey | Multiple methods | 238 | HIV + MSM |
| 69 | Y. Liu et al., 2015 | China | Qualitative-FGD | Convenience sampling | 60 | HIV+ & HIV-MSM |
| 70 | Logie, Newman, Chakrapani, & Shunmugam, 2012 | India | Quantitative-cross sectional survey | Convenience sampling | 200 | MSM |
| 71 | Logie, Newman, Weaver, Roungkraphon, & Tepjan, 2016 | Thailand | Quantitative-cross sectional survey | NGOs-driven sampling | 408 | Young MSM & transgender women |
| 72 | Lorway et al., 2011 | India | Qualitative-ethnographic, participant observation, community based study | NGOs-driven sampling | 10 | MSM as Community researchers |
| 73 | Lorway et al., 2014 | India | Qualitative-in-depth interview, community based study | Multiple methods | 70 | MSM |
| 74 | Ma et al., 2012 | China | Mixed-methods-cross sectional survey, in-depth interview, FGD | Multiple methods | 1178 | 1158 MSM & 20 key informants |
| 75 | Mburu et al., 2019 | Cambodia | Quantitative-cross sectional survey | RDS | 1375 | Transgender women |
| 76 | Mi et al., 2015 | China | Quantitative-trial | Multiple methods | 202 | HIV-positive MSM. |
| 77 | Mimiaga et al., 2015 | India | Qualitative-FGD, interview | NGOs-driven sampling | 55 | MSM & key informants |
| 78 | Mimiaga et al., 2013 | Vietnam | Qualitative-semi structured interview | NGOs-driven sampling | 23 | MSW |
| 79 | Mitchell et al., 2019 | Indonesia | Qualitative study-FGD, in-depth interview | Purposive sampling | 42 | Transgender women (waria) |
| 80 | Nehl et al., 2012 | China | Quantitative-cross sectional survey | RDS | 404 | MSM & money boys |
| 81 | Niu, Wang, Fang, Ip, & Lau, 2019 | China | Quantitative-cross sectional survey | Multiple methods | 336 | MSM |
| 82 | Niven, Jose, Rawstorne, & Nathan, 2018 | Timor Leste | Qualitative-interview | Purposive sampling | 15 | MSM & transgender women |
| 83 | Ojanen et al., 2019 | Thailand | Qualitative-life story interview | Purposive sampling | 19 | LGBTI identities |
| 84 | Oldenburg et al., 2014 | Vietnam | Quantitative-cross sectional survey | Multiple methods | 300 | MSWs |
| 85 | Pan et al., 2018 | China | Quantitative-cross sectional survey | RDS | 454 | MSM |
| 86 | Philbin et al., 2018 | Vietnam | Qualitative-in-depth interview, FGD | Multiple methods | 32 | MSM |
| 87 | Pongtriang, O’Brien, & Maguire, 2017 | Thailand | Qualitative-in-depth interview, field work observation | Consecutive sampling | 30 | MSM |
| 88 | Pratiwi, Waluyo, Yona, & Susanti, 2019 | Indonesia | Qualitative-phenomenological study, in-depth interview | Purposive sampling | 15 | HIV+ transgender women |
| 89 | Pyun et al., 2014 | China | Quantitative-cross sectional survey | Multiple methods | 318 | MSM |
| 90 | Ren et al., 2019 | China | Quantitative-cross sectional survey | Online recruitment | 521 | Gay men |
| 91 | Reyes et al., 2017 | The Philippines | Quantitative-cross sectional survey | Snowball sampling | 609 | LGBT |
| 92 | Robles & Canoy, 2019 | the Philippines | Qualitative-semistructured interview | Purposive sampling | 12 | HIV+MSM |
| 93 | Rongkavilit et al., 2015 | Thailand | Quantitative-trial | Random sampling | 74 | HIV + MSM |
| 94 | Sapsirisavat et al., 2016 | Thailand | Quantitative-cross sectional survey | Convenience sampling | 499 | MSM |
| 95 | Solomon et al., 2010 | India | Mixed-methods-FGD, cross sectional survey | RDS | 247 | Married MSM |
| 96 | Song et al., 2011 | China | Quantitative-cross sectional survey | Snowball sampling | 307 | Young migrant MSM (aged 18-29 y.o.) |
| 97 | Steward et al., 2013 | China | Qualitative-interview | Multiple methods | 30 | MSM |
| 98 | Su et al., 2018 | China | Quantitative-cross sectional survey | Multiple methods | 507 | MSM |
| 99 | Tangmunkongvorakul et al., 2013 | Thailand | Qualitative-in-depth interview, FGD | Convenience sampling | 46 | MSM |
| 100 | Tao et al., 2017 | China | Quantitative-cross sectional survey | Multiple methods | 367 | HIV + MSM |
| 101 | Thaker, Dutta, Nair, & Rao, 2018 | India | Quantitative-cross sectional survey | Convenience sampling | 225 | MSM & transgender women |
| 102 | Thi My Dung, Lee, Stewart, Thanh Nguyen, & Cuong Nguyen, 2016 | Viet Nam | Quantitative-cross sectional survey | RDS | 399 | MSM |
| 103 | Thomas, Mimiaga, Mayer, Closson, et al., 2012 | India | Qualitative-FGD, interview | Purposive sampling | 64 | 55 MSM & 9 key informants |
| 104 | Thomas, Mimiaga, Mayer, Perry, et al., 2012 | India | Quantitative-cross sectional survey | NGOs-driven sampling | 210 | MSM |
| 105 | Thompson et al., 2013 | India | Qualitative-semi structured interview, community based study | Purposive sampling | 39 | Transgender women (kothis) |
| 106 | Tomori et al., 2018 | India | Qualitative-FGD, in-depth interview | NGOs-driven sampling | 363 | MSM |
| 107 | Tsui & Lau, 2010 | China | Quantitative-cross sectional survey | Multiple methods | 566 | MSM |
| 108 | Veronese et al., 2019 | Myanmar | Qualitative-in-depth interview, FGD | NGOs-driven sampling | 60 | 18 MSM, 19 transgender women, & 23 key informants |
| 109 | Vu et al., 2017 | Vietnam | Quantitative-cross sectional, community based study | Convenience sampling | 622 | MSM |
| 110 | Vu et al., 2016 | Vietnam | Quantitative-cross sectional, community based study | Convenience sampling | 622 | MSM |
| 111 | C. Wei et al., 2016 | China | Quantitative-cross sectional survey | Convenience sampling | 523 | MSM |
| 112 | C. Y. Wei et al., 2014 | China | Qualitative-FGD | Purposive sampling | 49 | MSM |
| 113 | Wijngaarden et al., 2018 | The Philippines | Qualitative-case series | Purposive sampling | 12 | MSM |
| 114 | Willie, Chakrapani, Hughto, & Kershaw, 2017 | India | Quantitative-cross sectional survey | Convenience sampling | 299 | transgender women |
| 115 | Wilson et al., 2011 | Nepal | Qualitative-in-depth interview | Purposive sampling | 14 | Transgender women (Metis) |
| 116 | F. Wu et al., 2018 | China | Qualitative-in-depth interview | Purposive sampling | 26 | HIV+ MSM |
| 117 | Y. Wu et al., 2015 | China | Quantitative-cross sectional survey | Consecutive sampling | 184 | HIV+ MSM |
| 118 | J. Xu et al., 2010 | China | Quantitative-cohort prospective | Referral | 218 | HIV- MSM |
| 119 | W. Xu, Zheng, & Kaufman, 2018 | China | Quantitative-cross sectional survey | Multiple methods | 1100 | MSM |
| 120 | Wenjian Xu, Zheng, Wiginton, & Kaufman, 2019 | China | Quantitative-cross sectional survey | Multiple methods | 1100 | MSM |
| 121 | X. Xu, Sheng, Khoshnood, & Clark, 2017 | China | Quantitative-cross sectional survey | Convenience sampling | 277 | HIV+ MSM |
| 122 | H. Yan et al., 2019 | China | Quantitative-cross sectional survey | Convenience sampling | 347 | HIV+ MSM |
| 123 | Z. H. Yan et al., 2019 | China | Qualitative-FGD, interview | NGOs-driven sampling | 28 | 14 Transgender women & 14 key informants |
| 124 | J. P. Yang et al., 2018 | China | Quantitative-trial | Convenience sampling | 10 | HIV+ MSM |
| 125 | X. Yang, Mak, Ho, & Chidgey, 2017 | China | Quantitative-cross sectional survey | Consecutive sampling | 211 | HIV + MSM |
| 126 | Yi et al., 2018 | Cambodia | Quantitative-cross sectional survey | RDS | 1375 | transgender women |
| 127 | J. Zhang, Zheng, & Zheng, 2017 | China | Quantitative-cross sectional survey | Online recruitment | 584 | MSM |
| 128 | Zhao et al., 2015 | China | Quantitative-cross sectional survey | Snowball sampling | 1312 | MSM |
| 129 | Zhu, Liu, Chen, Zhang, & Qu, 2018 | China | Quantitative-cross sectional survey | Convenience sampling | 365 | MSM |
