# Supplementary material for: Methodologies of Stigma-Related Research Amongst Men Who Have Sex With Men (MSM) and Transgender People in Asia and the Pacific Low/Middle Income Countries (LMICs): A Scoping Review
Source: Front Reprod Health. 2021 Oct 29;3:688568. doi: 10.3389/frph.2021.688568 (PMC9580832; doi:10.3389/frph.2021.688568)
Supplement: Supplementary file 4 [file Table_4.DOCX]

Table 4

Table 4. Variables and measures used by study

| **No** | **Variable being measured** | **Measure/scale** | **Studies using this measure/scale** | **Country** | **Number of participants** | **The Cronbach’s Alpha/ Internal consistency** |
| --- | --- | --- | --- | --- | --- | --- |
| 1 | Mental health | The 5-item World Health Organization Well-Being Index (WHO-5) | Chan and Mak (2019) | China | 206 | 0.83 |
|  |  | The Thai General Health Questionnaire | Rongkavilit et al. (2015) | Thailand | 74 | 0.85 |
| 2 | Depression | The Centre for Epidemiological Studies Depression (CES-D 20).  A 20-item scale designed to measure depressive symptoms experienced by the patient within the past week | Wu et al. (2015) | China | 184 | 0.85 |
|  |  |  | Pan et al. (2018) | China | 454 | 0.88 |
|  |  |  | Yan et al. (2019) | China | 347 | 0.918 |
|  |  |  | Thomas et al. (2012) | India | 210 | NA |
|  |  |  | Deuba et al. (2013) | Nepal | 339 | NA |
|  |  |  | Du et al. (2018) | China | 321 | 0.853 |
|  |  |  | Mburu et al. (2019) | Cambodia | 1375 | 0.88 |
|  |  |  | Yi et al. (2018) | Cambodia | 1375 | 0.88 |
|  |  |  | Choi, Steward, Miege, and Gregorich (2017) | China | 493 | 0.84 |
|  |  |  | Wu et al. (2015) | China | 184 | 0.85 |
|  |  |  | Pan et al. (2018) | China | 454 | 0.88 |
|  |  |  | Lin et al. (2016) | China | 321 | 0.853 |
|  |  | The short-form Centre for Epidemiological Studies Scale (CES-D 12).  a 12-item questionnaire concerning depressive symptoms experienced in the past week | Huang et al. (2012) | China | 397 | 0.83 (money boys) and 0.85 (general MSM) |
|  |  |  | Nehl et al. (2012) | China | 404 | 0.85 |
|  |  | The short-form Centre for Epidemiological Studies Scale (CES-D 10).  A 10-item CES-D Depression scale to screen depressive symptomatology within the past week | Su et al. (2018) | China | 507 | 0.81 |
|  |  |  | Oldenburg et al. (2014) | Vietnam | 300 | NA |
|  |  |  | Wei et al. (2016) | China | 523 | 0.811 |
|  |  |  | Sapsirisavat et al. (2016) | Thailand | 499 | NA |
|  |  | The Beck Depression Inventory Fast-Screen(BDI-FS) | Chakrapani et al. (2017) | India | 300 | 0.91 |
|  |  |  | Logie, Newman, Chakrapani, and Shunmugam (2012) | India | 200 | 0.91 |
|  |  |  | Chakrapani, Willie, Shunmugam, and Kershaw (2019) | India | 300 | 0.83 |
| 3 | Depression and suicidal thoughts | Patient health questionnaire (PHQ-9) | J. P. Yang et al. (2018) | China | 10 | NA |
|  |  |  | Vu et al. (2017) | Vietnam | 622 | 0.80 |
|  |  | Adult Suicidal Ideation Questionnaire (ASIQ) | Reyes et al. (2017) | The Philippines | 609 | 0.99 |
| 4 | Anxiety and Depression | The Hospital Anxiety and Depression Scale (HADS) | Tao et al. (2017) | China | 367 | NA |
| 5 | Anxiety | the Self-Rating Anxiety Scale (SAS) | Wu et al. (2015) | China | 184 | 0.92 |
| 6 | Internalized stigma | The Self-Stigma Scale - Short Form (SSS-S) | Reyes et al. (2017) | The Philippines | 609 | 0.93 |
|  |  |  | Du et al. (2018) | China | 321 | 0.896 |
|  |  |  | J. Li, Mo, Wu, and Lau (2017) | China | 321 | 0.90 |
|  |  |  | Chong, Mak, Tam, Zhu, and Chung (2017) | China | 100 | 0.91 |
|  |  |  | Zhu, Liu, Chen, Zhang, and Qu (2018) | China | 365 | NA |
|  |  | The Self-Stigma Scale (SSS) | X. Yang, Mak, Ho, and Chidgey (2017) | China | 211 | 0.91 |
|  |  | The Internalized Stigma of AIDS Tool (ISAT) | X. Xu, Sheng, Khoshnood, and Clark (2017) | China | 277 | 0.83 |
|  |  | The Internalized Homophobia Scale | Ren et al. (2019) | China | 521 | 0.85 |
|  |  |  | Pyun et al. (2014) | China | 318 | 0.79 |
|  |  |  | W. Xu, Zheng, Wiginton, and Kaufman (2019) | China | 1100 | 0.89 |
|  |  |  | Su et al. (2018) | China | 507 | 0.65 |
| 7 | Transgender identity stigma | The transgender identity stigma scale (TGISS) | Willie, Chakrapani, Hughto, and Kershaw (2017) | India | 299 | NA |
| 8 | Gender non-conformity stigma | The Gender Non-Conformity Stigma Scale (GNCSS) | Chakrapani et al. (2017) | India | 300 | 0.84 |
|  |  |  | Logie et al. (2012) | India | 200 | 0.84 |
| 9 | Homosexual stigma | The Stigma Consciousness Scale | W. Xu et al. (2019) | China | 1100 | 0.76 |
| 10 | Shame and self-esteem | The Internalized Shame Scale | Brown, Low, Tai, and Tong (2016) | Malaysia | 234 | 0.98 |
| 11 | Self-esteem | the Rosenberg Self Esteem Scale | Yan et al. (2019) | China | 347 | 0.910 |
| 12 | Sexual compulsivity | The Sexual Compulsivity Scale | W. Xu et al. (2019) | China | 1100 | 0.92 |
| 13 | Sexual Sensation Seeking | the Sexual Sensation Seeking Scale | W. Xu et al. (2019) | China | 1100 | 0.88 |
| 14 | HIV Stigma | The HIV Stigma Revision Scale | Yan et al. (2019) | China | 347 | 0.925 |
|  |  | The HIV-related Stigma Assessment scale | Logie, Newman, Weaver, Roungkraphon, and Tepjan (2016) | India | 200 | 0.91 |
|  |  | The Steward’s HIV stigma scale | Tao et al. (2017) | China | 367 | 0.92 |
|  |  | The Berger’s HIV Stigma Scale | Z. Li, Hsieh, Morano, and Sheng (2016) | China | 266 | 0.95 |
| 15 | HIV/AIDS related stigma and discrimination | The HIV/AIDS related stigma and discrimination scale | Fan et al. (2016) | China | 391 | NA |
| 16 | AIDS-related Stigma | The AIDS-related Stigma Scale | Wei et al. (2016) | China | 532 | 0.805 |
| 17 | HIV stigma from the public | Perceived Devaluation-Discrimination Scale | Chan and Mak (2019) | China | 206 | 0.78 |
| 18 | HIV Knowledge | The 18-item HIV Knowledge Questionnaire | Rongkavilit et al. (2015) | Thailand | 74 | 0.60 |
| 19 | Disgust sensitivity (the pathogen disgust, sexual disgust, and moral disgust subscales) | Three Domain of Disgust Scale | Zhang, Zheng, and Zheng (2017) | China | 584 | 0.65, 0.66, and 0.71 |
| 20 | Social support | The social support scale | J. P. Yang et al. (2018) | China | 10 | NA |
|  |  | The Social Support Rating Scale (SSRS) | Liu et al. (2018) | China | 807 | 0.89– 0.94 |
|  |  |  | Yan et al. (2019) | China | 347 | 0.794 |
|  |  | Multi-dimensional Scale of Perceived Social Support (MSPSS) | Chakrapani et al. (2019) | India | 300 | 0.89 |
| 21 | Social provisions | the Weiss Social Provisions Scale | Nehl et al. (2012) | China | 404 | 0.84 |
| 22 | Stress | Perceived Stress Scale (PSS) | Reyes et al. (2017) | The Philippines | 609 | 0.70 |
|  |  | Gay Related Stressful Life Events Scale | Liu et al. (2018) | China | 807 | NA |
|  |  | The 5-item Mental Health Inventory | Chong et al. (2017) | China | 100 | NA |
| 23 | Mastery | the 7-item Mastery Scale | X. Xu et al. (2017) | China | 277 | 0.86 |
| 24 | Resilient coping | Ego-Resilience scale (ER89) | Yan et al. (2019) | China | 347 | 0.852 |
|  |  | The Brief Resilient Coping Scale (BRCS) | Logie et al. (2012) | India | 200 | 0.91 |
|  |  | The Button’s Identity Management Strategies Scale | Choi et al. (2017) | China | 493 | 0.86 |
| 25 | Motivational Readiness | The Readiness Ruler | Rongkavilit et al. (2015) | Thailand | 74 | 0.44 |
| 26 | Religious motivation | The Religious Orientation Scale | Brown et al. (2016) | Malaysia | 234 | 0.82 |
| 27 | Positive Affect and Negative Affect | Positive and Negative Affect Schedule (PANAS) scale | J. Li et al. (2017) | China | 321 | 0.86 and 0.93 |
| 28 | Alcohol use | Alcohol Use Disorder Identification Test scale (AUDIT-10) | Ha, Risser, Ross, Huynh, and Nguyen (2015) | Vietnam | 451 | 0.82 |
|  |  | The Alcohol Use Disorder and Associated Disabilities Interview Schedule IV (AUDADIS-IV) | Deuba et al. (2013) | Nepal | 339 | NA |
|  |  | The AUDIT-C brief alcohol screen | Aparna et al. (2018) | Vietnam | 205 | NA |
